# State Laws for the Regulation of the Practice of Medicine

**Published:** 1889-03

**Authors:** 


STATE LAWS FOR THE REGULATION OF THE PRACTICE OF MEDICINE.—In many States throughout the Union State examining boards and boards of health exist, having an eye to the protection of the people against quackery, and to the promotion of public sanitation, but after all it must be conceded that little good has been accomplished. Quackery holds it own, as does anti-prohibition in the liquor question. The difficulty lies in the fact that the moral sentiment of the masses is not yet ripe for reforms of this character.

